# Supplementary material for: Complex‐centric proteome profiling by SEC‐SWATH‐MS
Source: Mol Syst Biol. 2019 Jan 14;15(1):e8438. doi: 10.15252/msb.20188438 (PMC6346213; doi:10.15252/msb.20188438)
Supplement: Supplementary file 7 — Dataset EV6 [file MSB-15-e8438-s007.zip › feature_plots_bioplex/Q96C28.pdf]

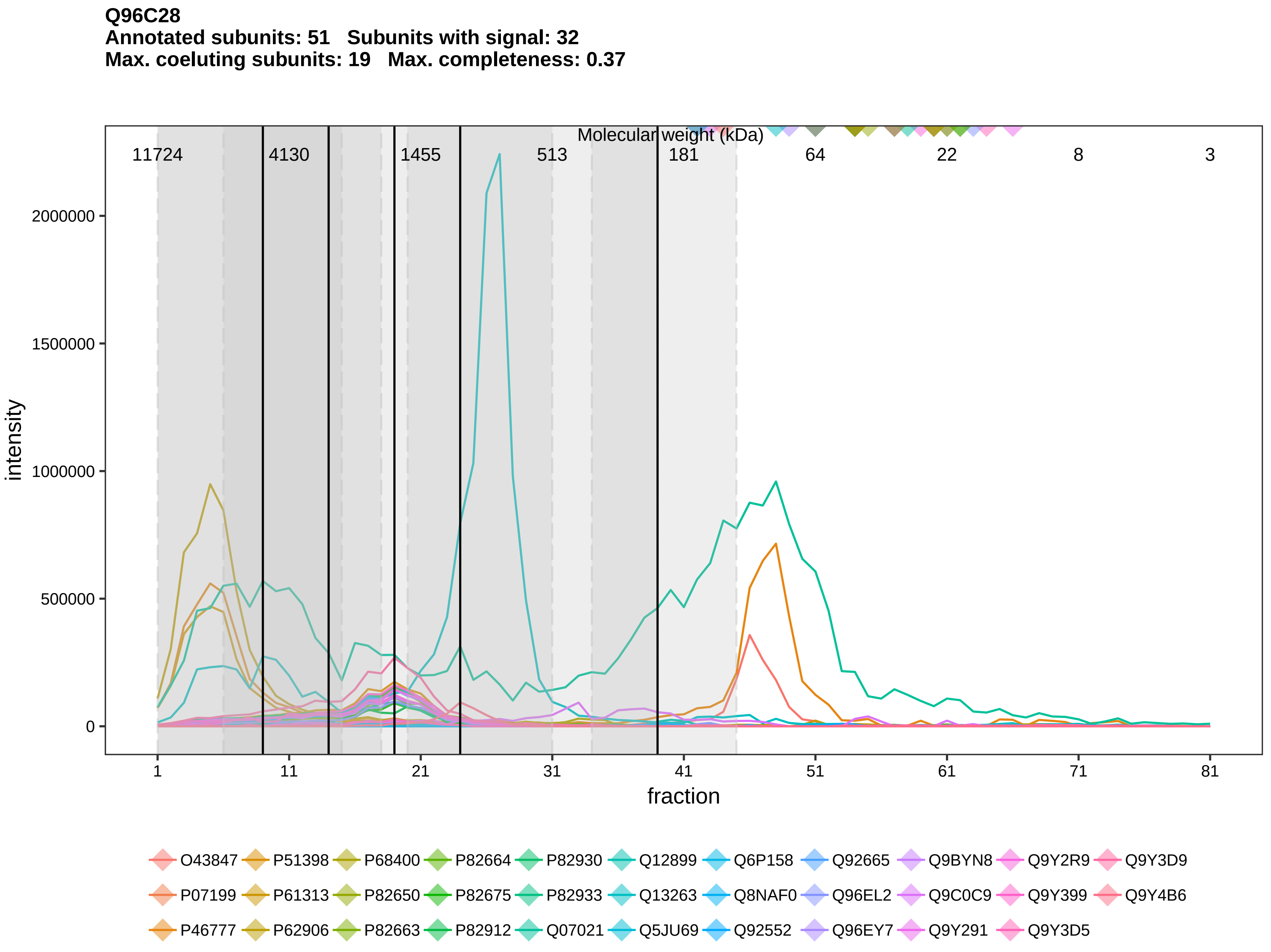

Q96C28
Annotated subunits: 51 Subunits with signal: 32
Max. coeluting subunits: 19 Max. completeness: 0.37
Molecular weight (kDa)
11724
4130
1455
513
181
64
22
8
3
intensity
2000000
1500000
1000000
500000
0
1
11
21
31
41
51
61
71
81
fraction
O43847
P07199
P46777
P51398
P61313
P62906
P68400
P82650
P82663
P82664
P82675
P82912
P82930
P82933
Q07021
Q12899
Q13263
Q5JU69
Q6P158
Q8NAF0
Q92552
Q92665
Q96EL2
Q96EY7
Q9BYN8
Q9C0C9
Q9Y291
Q9Y2R9
Q9Y399
Q9Y3D5
Q9Y3D9
Q9Y4B6
